# Effects of Spent Mushroom Substrate Treated with Plant Growth-Promoting Rhizobacteria on Blueberry Growth and Soil Quality

**DOI:** 10.3390/microorganisms13040932

**Published:** 2025-04-17

**Authors:** Mengjiao Wang, Desheng Sun, Zhimin Xu

**Affiliations:** 1 School of Biological Science and Engineering, Shaanxi University of Technology, Hanzhong 723000, China; 2 Collaborative Innovation Center for Comprehensive Development of Biological Resources in Qinling-Ba Mountains, Hanzhong 723000, China; 3 Sanqin Talents, Shaanxi Provincial First-Class Team, Contaminated Soil Remediation and Resource Utilization Innovation Team at Shaanxi University of Technology, Hanzhong 723000, China; 4 Shaanxi Scientific Instrument Service Center, Xi’an 710054, China; sunds0814@163.com; 5 School of Nutrition and Food Sciences, Louisiana State University, Baton Rouge, LA 70803, USA; zxu@agcenter.lsu.edu

**Keywords:** SMS, PGPR, blueberry, rhizosphere

## Abstract

Spent mushroom substrate (SMS) is the residual biomass generated after harvesting the fruitbodies of edible fungi. It is produced in large quantities and contains abundant nutrients. Plant growth-promoting rhizobacteria (PGPR) are a group of plant-associated microorganisms known for their ability to enhance plant growth, improve disease resistance, and boost soil quality. In this study, three PGPR strains with the highest plant growth-promoting potential were selected based on their ability to grow effectively in SMS extract. The SMS substrates were mixed with PGPR solutions and sterile water to establish a batch culture system. The mixture was initially incubated at 28 °C for 3 days, followed by continuous aerobic decomposition in a ventilated environment for 180 days. Based on the quality analysis of the PGPR-treated SMS, the 54-day treatment for transplanting blueberry seedlings was selected. The PGPR-treated substrates showed significantly higher TN, HN, and AP than controls (*p* < 0.05), suggesting a potential role of PGPR in enhancing nutrient availability. Alpha diversity index analysis revealed significant differences in microbial diversity between the PGPR-treated substrates and the control. Furthermore, the PGPR-treated substrates significantly influenced plant growth characteristics, soil nutrient content, and rhizosphere microbial diversity. Enhanced plant growth characteristics were strongly correlated with increased soil nutrient levels, suggesting a potential link between rhizospheric microbial communities and plant growth performance. This study provides a novel approach and experimental framework for the utilization of SMS and the development of PGPR-based biofertilizers, offering valuable insights into sustainable agricultural practices.

## 1. Introduction

Edible mushrooms are widely regarded as “next-generation food” owing to their high nutritional value and biological functionality [1]. Spent mushroom substrate (SMS), the residual biomass remaining after the harvest of edible fungal fruitbodies, is a significant byproduct of mushroom cultivation [2]. The production of 1 kg of fresh mushrooms yields approximately 5 kg of wet SMS [3]. Global production projections, derived from the 2022 baseline output (14.0 million metric tons) and accounting for a 4–6% annual growth rate (Global Mushroom Market Report, 2023), estimate 2024 yields at 15.0–15.5 million metric tons [4,5]. Consequently, the associated SMS production reaches 75.0–77.5 million metric tons annually, necessitating urgent development of valorization strategies to address environmental concerns from this lignocellulosic byproduct. The efficient recycling and valorization of SMS are critical for advancing the sustainable development of the mushroom industry according to the principles of a circular economy [6].

SMS is composed of residual mushroom mycelium and the growth substrate, which typically includes materials such as cottonseed hulls, wood chips, wheat bran, corn cobs, straw, and other cellulose-rich components [7]. This versatile byproduct offers a wide range of potential applications. For example, SMS has been employed as animal feed to enhance microbial balance and promote livestock growth [7]. Additionally, its unique properties—such as excellent air permeability, water and nutrient retention capabilities, and a loose texture—make it a promising candidate for use as a soil conditioner [8]. Moreover, due to its high lignocellulose content, SMS has been investigated as a feedstock for biofuel production, including biogas, ethanol, bio-oil, and solid fuels [9]. Notably, low-pressure hydrocarbons derived from SMS have been converted into carbon-absorbing microcapsules, demonstrating potential as adsorbents for post-combustion CO_2_ capture [10].

PGPRs are a group of plant-associated microorganisms known for their ability to enhance plant growth, improve disease resistance, and boost soil quality [11]. Owing to their beneficial traits—such as nitrogen fixation, phosphate and mineral solubilization, plant growth stimulation, and prevention of soil-borne diseases—PGPR strains have been widely utilized as microbial starters for fermenting industrial and agricultural waste [12]. For instance, waste proteins have been employed as additives in cultures of PGPR strains, such as *Bacillus amyloliquefaciens* SQR9, to produce high-quality bio-organic fertilizers [13]. Globally, approximately 5.1 billion tons of crop straw are produced annually. However, the open-field burning of this straw not only represents a significant waste of resources but also contributes to air pollution [14]. In this context, the application of PGPR for straw returning has been shown to enhance soil organic matter content and improve overall soil quality [15].

Building on evidence that raw SMS has good nutritional content, but is not easily broken down and utilized by plants, and PGPRs can promote plant growth and convert nutrients that are difficult for plants to use into nutrients that can be used by PGPR to enhance nutrient mineralization, we postulated that strain-specific decomposition would mitigate SMS toxicity while preserving growth-promoting elements. The 54-day threshold (observed in preliminary trials) would balance decomposition and nutrient retention, and 1:4 mixing ratio was adopted to maintain soil structure. After 45 days, the survival rate, plant height, and chlorophyll (Chl) content of the transplanted seedlings and the rhizosphere microenvironment of the blueberry seedlings were analyzed. All results were systematically analyzed to evaluate the impact of SMS decomposition on blueberry growth and soil improvement. The findings of this study provide a feasible research framework and novel insights for the application of SMS in agricultural practices.

## 2. Materials and Methods

### 2.1. Preparation of PGPR Strains and Detection of SMS Bacteria Toxicity

The SMS was air-dried naturally and ground into a fine powder using a Retsch Grindomix GM 200 at 200 rpm for 10 min. The SMS powder was then sieved through a 20-mesh screen (the mesh pore is 0.85 mm). Subsequently, 20 g of the sieved SMS powder was added to a sterile triangular flask containing 200 mL of distilled water. The flask was placed in a shaker and agitated at 28 °C for 24 h. The resulting extract was filtered through a 0.2 μm cell filter membrane to obtain a clear solution.

Based on preliminary laboratory screening of PGPR [16,17], we selected three strains with the highest plant growth-promoting potential for further experimentation in manuscript. Their plant growth-promoting capabilities are detailed in Table S1. Each of the three selected PGPR strains was aseptically inoculated into an SMS extract solution using sterile inoculation loops, followed by incubation at 28 °C with constant shaking at 180 rpm for 6 days. The optical density at 600 nm (OD_600_) of the PGPR-containing SMS extract was measured every 24 h. For each treatment, three independent biological replicates were maintained (*n* = 3), with each replicate consisting of a separately inoculated culture flask. At each time point, all three replicate flasks were measured individually, and the mean OD_600_ ± standard deviation was calculated for growth curve plotting. The control group followed identical replication procedures using beef extract peptone medium (Hopebio, HB9141, Qingdao, China) [18].

### 2.2. Quality Analysis of SMS Treated by PGPR at Room Temperature

All PGPR strains were first incubated in liquid beef extract peptone medium at 28 °C for 72 h. Sterilized SMS, PGPR culture, and sterile water were then mixed in a ratio of 1:3:10 (*w*:*v*:*v*) in 5 L sterile triangle bottles and cultured at 28 °C for 3 days. The mixture was subsequently transferred to a well-ventilated room-temperature environment (25 ± 2 °C, relative humidity 60 ± 5%) for a natural culture process. The initial day was recorded as day 0 of the culture process. During this period, the material was periodically turned (every 7 days) to ensure aerobic conditions. These substrates for different strains were designated as T1, T2, and T3, where T1 represents strain T1, T2 represents strain T2, and T3 represents strain T3. The pH, organic carbon content (OC), total nitrogen content (TN), hydrolysable nitrogen content (HN), total phosphorus content (TP), available phosphorus content (AP), total potassium content (TK), and available potassium content (AK) of the SMS starter cultures were analyzed on days 3, 9, 27, 54, 90, 135, and 180 of culture process. The sampling time points (3, 9, 27, 54, 90, 135, 180 days) were strategically selected to capture initial microbial growth dynamics, including the peak PGPR activity observed at Day 3, while subsequent time points monitored key physicochemical transitions during decomposition, such as lignin degradation onset. The extended sampling through Day 180 allowed evaluation of final substrate stability, with this logarithmic distribution optimally representing nonlinear decomposition kinetics while aligning with standard composting monitoring intervals. The pH was measured using a pH meter, while OC, TN, HN, TP, AP, TK, and AK were determined using the methods described by Wang et al. [17]. OCC was determined by the potassium dichromate oxidation method (Walkley-Black procedure), TNC was measured using the micro-Kjeldahl digestion method, TPC was analyzed by flow injection analysis (AutoAnalyzer 3, Bran+Luebbe, Hamburg, Germany) following nitric-perchloric acid digestion, and TK was quantified via inductively coupled plasma optical emission spectrometry (ICP-OES; ICPS-7500, Shimadzu, Kyoto, Japan) [17]. Available nutrient fractions were assessed for HN by alkaline hydrolysis diffusion method, AP by molybdenum blue colorimetry (Olsen method), and AK by ammonium acetate extraction-flame photometry [19]. For each treatment combination, three independent biological replicates were established by inoculating separate culture flasks (*n* = 3). At each sampling time point, sterile aliquots were collected from all replicate flasks for elemental analysis. Measurements of nutritive element contents and pH were performed in triplicate for each biological replicate. To assess the baseline of the culture process, parallel control treatments were maintained under identical conditions. For parallel controls, only nutrient soil (Brighten 2024, a commercial peat-based substrate with pH 5.5–6.0, Pindstrup Group, Ryomgård, Denmark) was labeled TC1, and sterilized SMS mixed with nutrient soil at a 1:4 ratio was labeled TC2. The pH, OC, TN, HN, TP, AP, TK, and AK of the SMS starter cultures were analyzed on days 3, 9, 27, 54, 90, 135, and 180 of culture process. Data are presented as mean values ± standard deviation across biological replicates.

### 2.3. Transplanting of Blueberry Seedlings and Analysis of Growth Status

**Figure 1:**
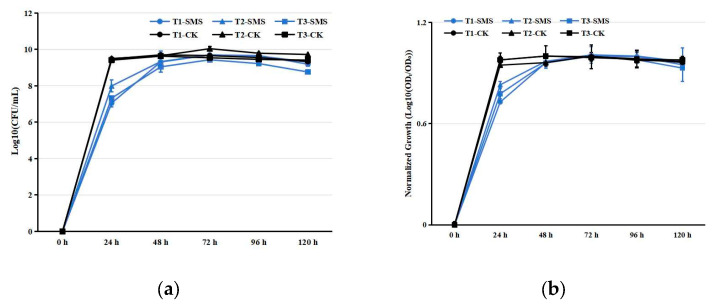
Growth kinetics of PGPR strains in different media. (**a**) Absolute growth curves (Log10-transformed OD_600_). (**b**) Normalized growth profiles (Log10(ODt/OD0)). Notes: T1, T2, T3 represent strain identifiers; SMS: spent mushroom substrate extract; CK: control medium (beef extract peptone). Data represent mean ± SD (*n* = 3).

Based on the quality analysis results of SMS treated by PGPR, the PGPR strains were incubated in liquid beef extract peptone medium at 28 °C for 72 h. Newly sterilized SMS, PGPR culture, and sterile water were then mixed in a ratio of 1:3:10 (*w*:*v*:*v*) in 5 L sterile triangle bottles and cultured at 28 °C for 3 days. The mixture was then transferred to a cool, ventilated environment at room temperature to continue the culture process. Based on preliminary nutrient analysis showing peak nitrogen and phosphorus availability at this stage (Figure 1), the PGPR-treated SMS was harvested after 54 days of decomposition. The treated substrate was then mixed with nutrient soil (Brighten 2024, a commercial peat-based substrate with pH 5.5–6.0, Pindstrup Group, Ryomgård, Denmark) at a 1:4 ratio to prepare the cultivation substrate, which was then transferred into pots (13.72 dm^3^). These substrates for different strains were designated as T1, T2, and T3, where T1 represents strain T1, T2 represents strain T2, and T3 represents strain T3. For controls, nutrient soil alone (labeled TC1) and sterilized SMS mixed with nutrient soil at a 1:4 ratio (labeled TC2) were also placed in identical pots (13.72 dm^3^). Fifty-day old, healthy blueberry seedlings, approximately 5 cm in height and previously cultivated in the same nutrient soil, were selected for the transplanting experiment. For each treatment (T1, T2, T3, TC1, TC2), three replicate pots were prepared. Each pot was planted with 25 blueberry seedlings. All pots were arranged in completely randomized positions within the controlled-environment growth chamber, with weekly rotation to minimize microenvironmental effects. The growth chamber was under the following conditions. Environmental Control: temperature, 25 ± 1 °C (mean ± SD); relative humidity, 70 ± 5%; CO_2_ concentration, 400 ± 50 ppm; lighting conditions, white LED arrays (photosynthetically active radiation/PAR: 400–700 nm), photon flux density of 120 μmol photons m^−2^ s^−1^ (measured at canopy height with quantum), and continuous illumination (~1500 Lx); and the irrigation protocol was as needed based on substrate moisture. No fertilizer is applied during cultivation.

The survival rate of the blueberry seedlings was recorded 45 days after transplantation. The survival rate was calculated as (%) = (Number of surviving seedlings)/25 × 100 (%).The number of initial blueberry seedlings transplanted per pot was 25. There were three pots for every treatment. Plant height was measured on day 45 as the distance from the base of the plant to the tip of the main shoot for all blueberry seedlings that survived [20]. The eighth leaf from four selected seedlings with 10 cm height in each treatment was harvested, and the total chlorophyll (Chl) content was determined using the Lichtenthaler method [21].

### 2.4. Analysis of Nutrient Element Contents and Microbial Diversity in Blueberry Seedlings Rhizosphere Soil

Following leaf harvesting, rhizosphere soil samples were collected using the method described by Wang et al. [16]. For each treatment, rhizosphere soil from all seedlings within each pot was collected, thoroughly homogenized, and then divided into two aliquots: one for elemental analysis and the other for microbial diversity assessment. The contents of nutrient elements were analyzed according to the methodology outlined in Section 2.2.

High-throughput amplicon sequencing was employed to analyze microbial diversity in the rhizosphere soil of blueberry seedlings. Genomic DNA was extracted using the Fast DNA Spin Kit for Soil (MP Biomedicals, Santa Ana, CA, USA). Specific primers (515F/806R for bacterial 16S rDNA [22] and ITS3-F/ITS4R for fungal ITS regions [23]; Invitrogen, Carlsbad, CA, USA) were used to amplify the V4 region of bacterial 16S rDNA and the ITS4 region of fungal DNA. A PCR amplification was performed using a Bio-Rad S1000 thermal cycler (Bio-Rad Laboratories, Hercules, CA, USA) with the following thermocycling program: initialization at 94 °C for 5 min; 30 cycles of denaturation at 94 °C for 30 s, annealing at 52 °C for 30 s, and extension at 72 °C for 30 s; followed by a final elongation at 72 °C for 10 min, according to the protocol described by Wang et al. [19]. The amplification purified PCR products (290–310 bp for 16S and 370–450 bp for ITS) were used to construct DNA libraries using NEBNext Ultra DNA Library Prep Kits (New England Biolabs, Ipswich, MA, USA). Sequencing was carried out on the Illumina HiSeq 2500 platform (Illumina, San Diego, CA, USA).

Operational taxonomic units (OTUs) were assigned to represent species, and taxonomic annotation was performed using the Silva database (version 138.1, released 10 December 2020) (https://www.arb-silva.de/ ((accessed on 14 January 2025)). Raw sequence data were deposited in the Sequence Read Archive (https://submit.ncbi.nlm.nih.gov/subs/sra/ (accessed on 4 April 2025)), and accession numbers were obtained. Microbial diversity was evaluated using alpha and beta diversity indices, the Unifrac distance and principal coordinates analysis (PCoA) using QIIME 2 (Version 1.9.1, https://qiime2.org/ ((accessed on 14 January 2025))), and the results were visualized with the ggplot2 package (Version 2.15.3) in R (v4.1.0) [24,25].

### 2.5. Statistical Analysis

All experiments were conducted in triplicate and shown as mean value ± standard deviation, SD. If data presentation is required to indicate significant differences. Different lowercase letters were used to indicate significant differences (Tukey’s HSD test, *p* < 0.05). Relationships between the growth-promoting characteristics of PGPR strains and plant growth parameters, soil nutrient content, and rhizosphere soil microbial diversity were evaluated using Pearson’s correlation analysis, Kendall’s correlation analysis, and Spearman’s correlation analysis. The integrating principal component analysis (PCA) and other data analysis used IBM SPSS Statistics (v28.0, IBM Corp., Armonk, NY, USA). Correlation heatmap was drawn using EXCEL based on the calculation results of Pearson’s correlation analysis.

## 3. Results

### 3.1. Assessment of SMS Bacterial Toxicity and Quality Analysis of SMS Treated by PGPRs at Room Temperature

PGPRs inoculated in SMS extract exhibited normal growth (Figure 1). The cell counts of each PGPR strain reached their peak 72 h after inoculation in the SMS extract (Figure 1). In contrast, the cell counts of PGPRs in beef extract peptone liquid medium peaked at 48 h post-inoculation (Figure 1). Although the growth rates of PGPRs in the SMS extract were slightly slower compared to the control group, the peak cell counts were comparable. Statistical analysis of the SMS decomposition substrate revealed significant treatment effects on key parameters (Table S2). Under the conditions of PGPR treatment, the elemental composition exhibited distinct temporal patterns over the 180-day observation period. OC showed an initial accumulation phase, peaking at 435.89 g/kg by day 54 before slightly declining to 428.04 g/kg (Tables S2–S4). Nitrogen dynamics were marked by a rapid early surge in HN, which increased 237% within the first 3 days, while TN displayed a more gradual rise followed by stabilization (Tables S2–S4). Phosphorus fractions demonstrated contrasting behaviors; TP nearly doubled by day 90, whereas AP initially decreased before recovering to exceed baseline levels (Tables S2–S4). Potassium availability progressively improved, with AK increasing by 39% by mid-period, despite a modest 8% decline in TK (Tables S2–S4). The system maintained remarkable pH stability throughout, fluctuating minimally between 5.50 and 5.78 (Tables S2–S4). For TC1, all measured parameters, including OC (318.90–323.40 g/kg), TN (7.99–8.03 g/kg), HN (0.47–0.49 g/kg), TP (1.18–1.21 g/kg), AP (24.77–26.15 mg/kg), TK (13.01–13.19 g/kg), and AK (0.20–0.21 g/kg), remained statistically stable (*p* > 0.05), with no significant temporal variations observed (Table S5). Similarly, pH values fluctuated minimally between 5.50 and 5.61 throughout the incubation. In contrast, TC2 exhibited slightly higher but equally stable nutrient concentrations (OC: 345.21–354.33 g/kg; TN: 8.10–8.33 g/kg; HN: 0.51–0.53 g/kg; TP: 1.49–1.58 g/kg; AP: 534.50–573.44 mg/kg; TK: 13.02–13.89 g/kg; AK: 0.27–0.30 g/kg), with pH ranging from 5.60 to 5.74 (Table S6). Both treatments maintained nutrient stability, demonstrating that neither strain induced significant nutrient depletion or accumulation during the half-year incubation period.

### 3.2. Analysis of Growth Performance of Blueberry Seedlings

**Figure 2:**
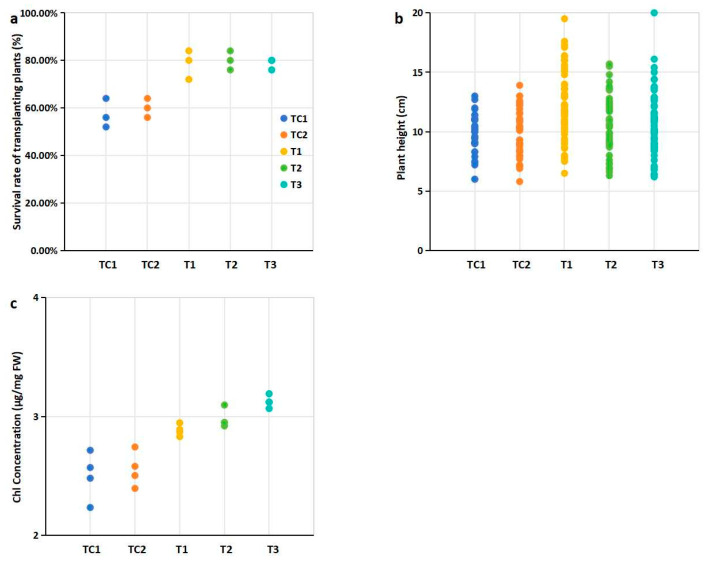
Effects of PGPR inoculation on physiological indices of blueberry plants: (**a**) Survival rates of transplanted plants after 45 days; (**b**) Plant height (cm); (**c**) Chlorophyll concentration (total Chl, mg/g FW, fresh weight). Notes: Survival (%) = (Number of surviving seedlings)/25 × 100 (%). 25 represents the initial number of blueberry seedlings transplanted per pot, and there were three pots for every treatment. Plant height was measured on day 45 for all blueberry seedlings survived. The eighth leaf from four selected seedling with 10 cm height in each treatment was harvested. Note: Dots of the same color represent biological repetitions of the same treatment. The treatment represented by the color of the points in figures b and c is consistent with that in Figure a.

The survival rates of all blueberry seedlings exceeded 58%. Compared to the control group, the survival rates of seedlings transplanted into cultivation media supplemented with PGPR-treated substrates and nutrient soil were significantly higher (Figure 2a). Notably, the highest survival rate (80.76%) was observed in seedlings grown in the medium mixed with T2 strain-treated substrates and nutrient soil (Figure 2a, Table S7). In terms of plant height, seedlings transplanted into cultivation media containing PGPR-treated substrates and nutrient soil generally exhibited greater height compared to the control group. Among these, the seedlings grown in the medium mixed with T1 strain-treated substrates showed a statistically significant difference in plant height relative to the control (Figure 2b). Additionally, significant differences in chlorophyll (Chl) content were observed between the treatment groups and the control group (Figure 2c).

### 3.3. Analysis of Nutrient Elements in Rhizosphere Soil

**Figure 3:**
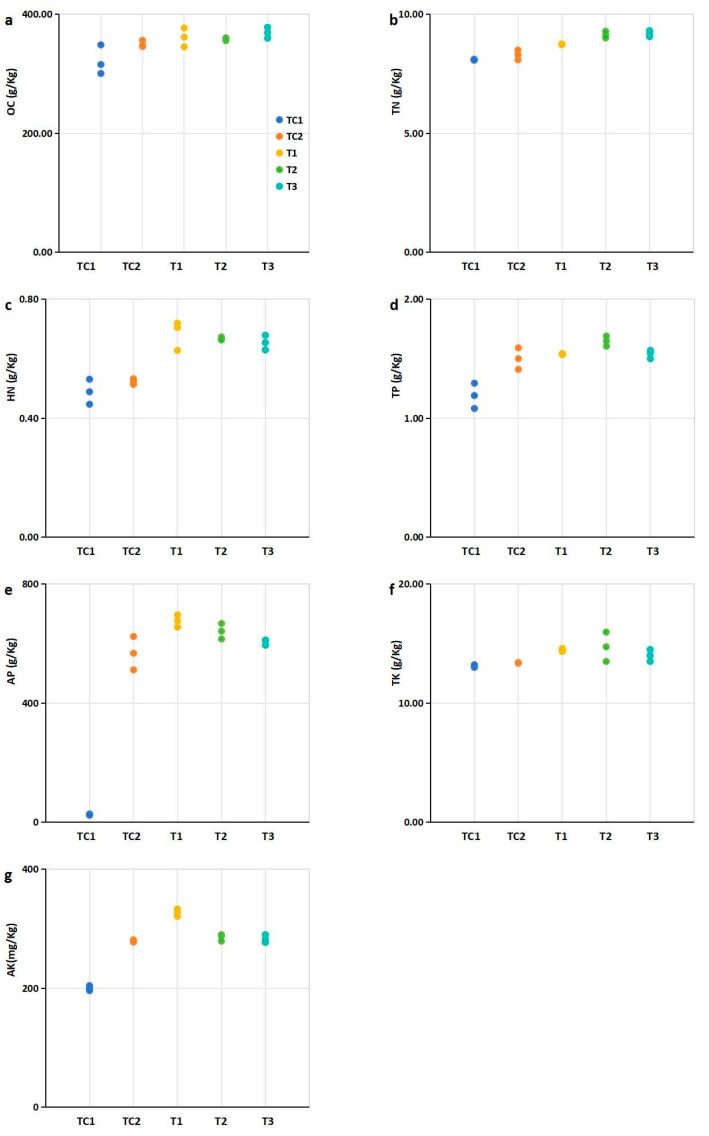
Nutritional element contents in rhizosphere soils of blueberry plants under different treatments. Experimental groups: PGPR-inoculated (T1, T2, T3) Control group: Nutrient soil alone (labeled TC1) and sterilized SMS mixed with nutrient soil at a 1:4 ratio (labeled TC2). (**a**) Organic carbon (OC). (**b**) Total nitrogen (TN) contents; (**c**) Hydrolysable nitrogen (HN); (**d**) Total phosphorus (TP); (**e**) available phosphorus (AP); (**f**) Total potassium (TK); (**g**) Available potassium (AK). Notes: Data expressed three replicates per treatment. Abbreviations: OC, organic carbon; TN, total nitrogen; HN, hydrolysable nitrogen; TP, total phosphorus; AP, available phosphorus; TK, total potassium; AK, available potassium. Note: Dots of the same color represent biological repetitions of the same treatment. The treatment represented by the color of the points in (**b**,**c**) is consistent with that in (**a**).

The nutrient element contents—including organic carbon content (OC), total nitrogen content (TN), total phosphorus content (TP), total potassium content (TK), hydrolysable nitrogen content (HN), available phosphorus content (AP), and available potassium content (AK)—were analyzed in the blueberry rhizosphere cultivation medium after leaf harvesting (Figure 3).

Compared to the control (TC1, nutrient soil alone), the nutrient element contents in the rhizosphere cultivation substrates mixed with SMS were significantly higher, with the exception of TK (Figure 3). Specifically, the TN, HN, and AP contents in substrates mixed with PGPR-treated SMS were significantly higher than those in substrates containing untreated SMS (Figure 3b,d). Additionally, the TP content in substrates mixed with T2-treated SMS was significantly higher than in untreated SMS mixtures (Figure 3c). Similarly, the TK content in substrates mixed with T1- and T2-treated SMS was significantly higher than in untreated SMS mixtures (Figure 3e). Furthermore, treatment of the T1 strain significantly increased the AK content in the rhizosphere cultivation substrates compared to untreated SMS mixtures (Figure 3f).

### 3.4. Analysis of Rhizosphere Soil Microbial Community

**Figure 4:**
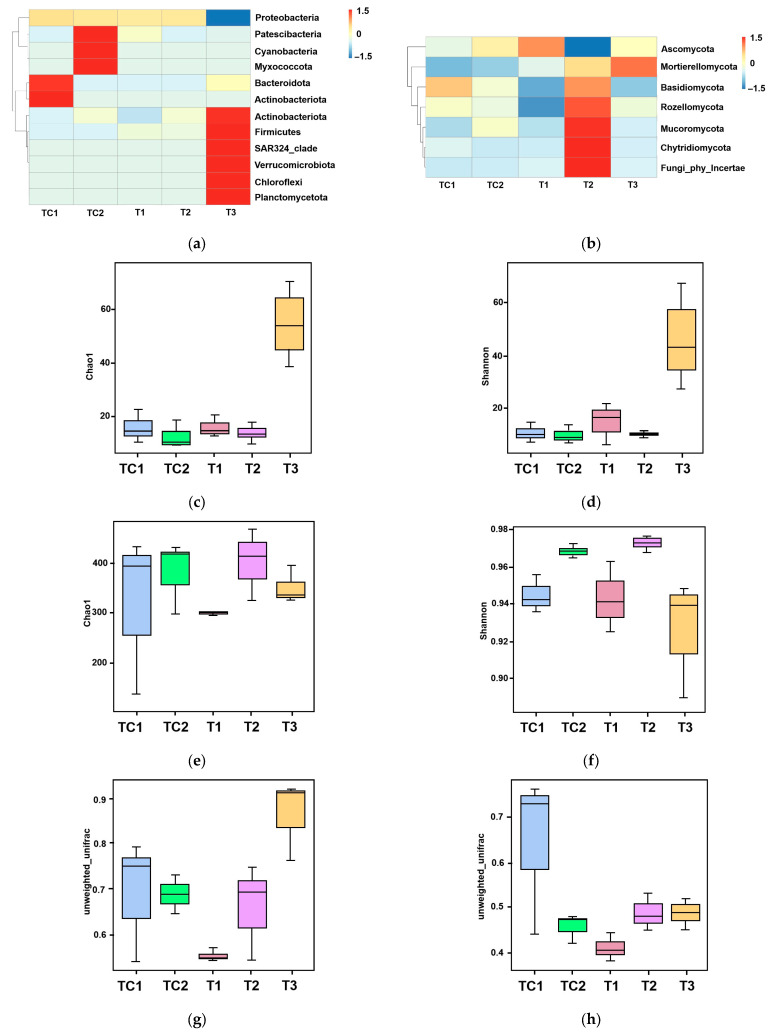

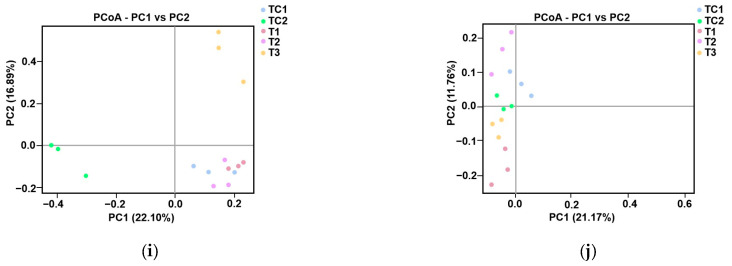
Microbial community analysis in blueberry rhizosphere. (**a**) Phylum-level bacterial composition heatmap. (**b**) Phylum-level fungal composition heatmap. (**c**,**d**) Bacterial α-diversity assessed using Chao1 (species richness) and Shannon (diversity) indices. (**e**,**f**) Fungal α-diversity assessed using Chao1 (species richness) and Shannon (diversity) indices. (**g**,**h**) Phylogenetic β-diversity based on unweighted UniFrac distances. (**i**,**j**) Principal coordinates analysis (PCoA) of bacterial (**i**) and fungal (**j**) communities.

High-throughput sequencing technology was employed to analyze the microbial diversity in the rhizosphere of blueberry seedlings. The sequence data were deposited as submission ID SUB15231282 and BioProject ID PRJNA1245621. Figure 4a presents the phylum-level bacterial community composition across five samples (TC1, TC2, and T1–T3). Analysis revealed 12 major bacterial phyla with distinct distribution patterns among samples. The control sample TC1 showed significant enrichment of Proteobacteria (Figure 4a). In contrast, experimental samples (T1–T3) exhibited variable abundances of Myxococcota and Bacteroidota (ranging from 0 to to 1.5) (Figure 4a). Notably, SAR324_clade and Verrucomicrobiota were consistently underrepresented (values ≤ 0) in all samples (Figure 4a). Firmicutes and Chloroflexi maintained stable populations (0) across most samples, while Planctomycetota demonstrated minimal compositional variation (Figure 4a). The phylum-level fungal distribution across five samples (control groups TC1–TC2 and experimental groups T1–T3) via a heatmap was illustrated in Figure 4b. Six major fungal phyla were identified, with Ascomycota dominating across all samples (Figure 4b). Experimental samples exhibited dynamic shifts; Basidiomycota and Mucoromycota displayed fluctuating abundances, while Rozellomycota and Chytridiomycota showed sporadic detection (Figure 4b). Mortierellomycota maintained stable representation, whereas unclassified fungi were consistently underrepresented (Figure 4b).

The α-diversity patterns of bacterial and fungal communities were analyzed using Chao1 (species richness) and Shannon (diversity) indices. For bacterial communities (Figure 4c,d), both indices showed progressive declines from T1 to T3, with Chao1 values indicating reduced species richness and Shannon indices (T1: 60; T2: 40; T3: 20) demonstrating parallel decreases in diversity. Fungal communities (Figure 4e,f) exhibited similar trends, with Chao1 values decreasing sharply from T1 (400) to T3 (200), while Shannon indices revealed a consistent diversity reduction across all samples (TC1: 0.98; TC2: 0.96; T1: 0.94; T2: 0.92; T3: 0.90).

Phylogenetic β-diversity was evaluated using unweighted UniFrac distance metrics (Figure 4g,h), which measure community dissimilarity based on taxa presence/absence patterns. Analysis revealed significant structural differences between control (TC1, TC2) and experimental (T1–T3) groups (Figure 4e,f). The control samples maintained intermediate dissimilarity values (UniFrac range: 0.6–0.7), while experimental samples displayed a progressive decrease in phylogenetic distance from T1 (0.5) to T3 (0.4) (Figure 4g,h).

Principal coordinates analysis (PCoA) was performed to evaluate species complexity (Figure 4i,j). In the PCoA plot, samples located closer to each other exhibited more similar microbial communities. For bacterial communities, a notable separation was observed between samples treated with strain T3, those treated with CKP, and the other three samples (Figure 4i). For fungal community composition, a clear separation was observed between PGPR-treated samples and the control samples (Figure 4j).

### 3.5. Correlations Between Growth-Promoting Characteristics of PGPR Strains and Plant Growth, Soil Element Content, and Rhizosphere Microbial Diversity

**Figure 5:**
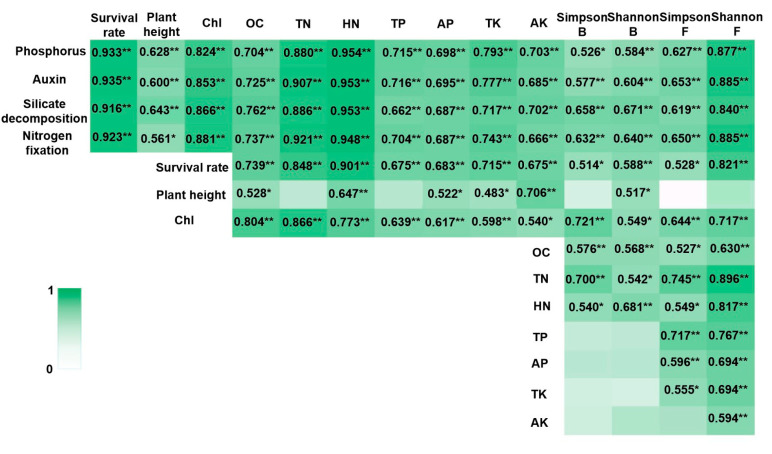
Correlation heatmap integrating PGPR traits, plant growth, soil elements, and rhizosphere microbiota. Note: Chl: total chlorophyll, OC: The organic carbon contents, TN: total nitrogen contents, HN: hydrolysable nitrogen contents, TP: total phosphorous contents, AP: available phosphorous contents, TK: total potassium contents, and AK: available potassium contents. Simpson B and Shannon B were α diversity index of rhizosphere bacteria, and Simpson F and Shannon F were α diversity index of rhizosphere fungi. ** was as significantly associated at 0.01 level (bilateral). * was as significantly associated at 0.05 level (bilateral).

Pearson’s correlation analysis revealed that the plant growth-promoting capabilities of PGPR strains were highly significantly correlated with plant growth characteristics, soil nutrient element contents, and microbial diversity in the blueberry rhizosphere cultivation substrates (Figure 5). Soil element content showed a highly significant correlation with the survival rates of blueberry seedlings (Figure 5). Among the soil elements, only hydrolysable nitrogen content (HN) and available potassium content (AK) exhibited extremely significant correlations with the plant height of blueberry seedlings (Figure 5). All soil element contents, except AK, demonstrated highly significant correlations with the chlorophyll (Chl) content in blueberry seedling leaves (Figure 5). Additionally, the survival rates of blueberry seedlings were highly significantly correlated with the Shannon index of rhizosphere soil microbial diversity (Figure 5). The Simpson index for bacteria, as well as the Simpson and Shannon indices for fungi, showed highly significant correlations with the Chl content of blueberry seedling leaves (Figure 5). Furthermore, significant correlations were observed between soil element contents and the Shannon index of fungal diversity (Figure 5). The results of Kendall’s correlation analysis and Spearman’s correlation analysis were consistent with the above results (Tables S8–S13).

The PCA results revealed three main principal components, as detailed in Table S14. These components exhibited strong loadings for the following variables: phosphorus (0.961), auxin (0.970), silicate decomposition (0.965), nitrogen fixation (0.970), survival rate (0.924), chlorophyll (Chl) content (0.867), total nitrogen content (TN) (0.929), hydrolyzable nitrogen content (HN) (0.950), and the Shannon diversity index (Shannon *p*) (0.893).

## 4. Discussion

### 4.1. Detection of SMS Bacterial Toxicity and Quality Analysis of SMS Treated by PGPRs at Room Temperature

The nutrient-rich byproducts of mushroom cultivation contain valuable growth substrates, including residual proteins, polysaccharides, and mineral complexes [26]. Recent studies have characterized SMS as a heterogeneous matrix where nutrient bioavailability depends on fungal species and composting protocols [27]. In this study, all tested PGPR strains exhibited typical growth kinetics in this medium, reaching maximum cell densities after 72 h of incubation (Figure 1). The observed growth patterns demonstrate efficient utilization of available nutrients without apparent metabolic inhibition, suggesting excellent compatibility between PGPR physiological requirements and the nutritional profile of the cultivation byproduct. These results substantiate recent findings on PGPR metabolic versatility [14,28], while highlighting the potential of this agricultural byproduct as a sustainable culture matrix for biofertilizer development.

During the culture process, the concentrations of nutrient elements initially decreased as they were utilized to support PGPR growth. The increases in hydrolysable nitrogen (HN) and available phosphorus (AP) (Tables S3–S7) are consistent with known PGPR-mediated nutrient transformations [16,17], though multiple mechanisms could contribute. Considering the specific growth requirements of blueberry plants and soil quality standards [29], a 54-day treated culture substrate was selected and mixed with nutrient soil at a ratio of 1:4 to prepare the cultivation substrate.

### 4.2. PGPR-Treated Substrates Promote the Growth of Transplanted Blueberry Seedlings

Plant growth-promoting rhizobacteria (PGPR) enhance the adaptability of transplanted plants to natural environments [30]. In this study, evaluated PGPR may play a critical role in improving the survival rates of transplanted blueberry seedlings. Seedlings transplanted into PGPR-enhanced substrates exhibited significantly higher survival rates compared to controls (*p* < 0.05, Figure 2a), demonstrating the practical efficacy of this approach for improving survival rates.

Plant height, an important agronomic trait linked to crop yield and quality, is influenced by factors such as gibberellins and rhizosphere soil nutrient content [31]. Notably, taller blueberry seedlings were observed in cultivation substrates mixed with PGPR-treated substrates and nutrient soil. A significant difference in plant height was observed for seedlings transplanted into substrates mixed with T1 strain-treated substrates (Figure 2b), suggesting a potential association between PGPR-mediated substrate modification and improved plant growth.

Chlorophyll (Chl) content, a key parameter in agronomy and plant biology research, serves as a direct indicator of plant growth [32]. Significantly higher Chl levels were detected in leaves from blueberry seedlings grown in substrates mixed with PGPR-treated substrates (Figure 2c). The elevated chlorophyll levels (Figure 2c) likely reflect improved nutrient availability and optimized rhizosphere conditions mediated by PGPR activity. This is consistent with the known roles of PGPRs in increasing chlorophyll contents [33].

### 4.3. PGPR-Treated Substrates Improve the Properties of Cultivation Medium for Soil Maintenance

The nutrient content in the plant rhizosphere is a critical environmental factor closely associated with plant growth [34]. PGPR have been shown to enhance the nutrient content of rhizosphere soil through organic matter decomposition and nutrient solubilization [35]. In this study, The integration of PGPR-treated SMS into cultivation substrates was correlated with synergistic nutrient–microbe interactions (Figure 3, Figure 4 and Figure 5). While these changes are consistent with PGPR-mediated processes [36,37], the relative contributions of inoculated bacteria versus native microbiota remain to be quantified through controlled sterilization-inoculation experiments. Collectively, these effects (nutrient release coupled with microbiome remodeling) highlight the potential of SMS-PGPR composites as sustainable, multifunctional alternatives to conventional fertilizers in blueberry production.

The nutrient content in the plant rhizosphere is a critical environmental factor closely associated with plant growth [34]. PGPR have been shown to enhance the nutrient content of rhizosphere soil through organic matter decomposition and nutrient solubilization [35]. In this study, the integration of PGPR-treated SMS into blueberry cultivation substrates significantly elevated key nutrient levels compared to controls (Figure 3). Notably, substrates amended with PGPR-treated SMS exhibited marked increases in TN (18–22%), HN (15–20%), and AP (25–30%) relative to untreated SMS (Figure 3b,d), consistent with the documented role of PGPR in nitrogen fixation and phosphate solubilization [36,37]. The T2 strain further enhanced TP (12% increase; Figure 3c), while T1 and T2 treatments improved TK (8–10%; Figure 3e) and AK (15%; Figure 3f), underscoring strain-specific nutrient mobilization capabilities.

These nutrient dynamics were paralleled by PGPR-driven remodeling of the rhizosphere microbiome. High-throughput sequencing revealed pronounced shifts in bacterial and fungal community structure (Figure 4a,b). The depletion of dominant Proteobacteria in controls (TC1) and the enrichment of Myxococcota and Bacteroidota in PGPR-treated samples (T1–T3) (Figure 4a) suggest competitive exclusion or niche modification by inoculated strains. Fungal communities similarly shifted, with Ascomycota dominance and dynamic fluctuations in Basidiomycota and Mucoromycota abundances (Figure 4b), potentially linked to PGPR-mediated suppression of phytopathogens or cross-kingdom signaling [38].

The α-diversity trends—declining Chao1 and Shannon indices from T1 to T3 for both bacteria and fungi (Figure 4c–f)—indicate PGPR-induced selection pressure, likely favoring taxa with synergistic relationships (e.g., nutrient cyclers). Phylogenetic β-diversity analysis (UniFrac metrics; Figure 4g,h) and PCoA (Figure 4i,j) further confirmed the divergence of PGPR-treated communities from controls, with T3 strain eliciting the most distinct bacterial assemblage (PCoA axis 1: 40% variance). This aligns with prior observations of PGPR-driven microbiome specialization [39].

### 4.4. Correlations Between Growth-Promoting Characteristics of PGPR Strains and Plant Growth, Soil Nutrient Content, and Rhizosphere Microbial Diversity

The plant rhizosphere represents a highly complex microenvironment that plays a critical role in plant growth and development [40]. Interactions between plants and soil microenvironment factors, such as the rhizosphere microbial community and soil nutrient dynamics, predominantly occur within the rhizosphere [41]. Understanding these plant-microbe interactions is essential for enhancing crop yield and quality [42]. PGPRs confer multiple benefits to plants, including promoting microbial activity, enhancing soil fertility, and supporting plant growth [43]. Our correlation analyses revealed that the growth-promoting characteristics of PGPR strains were significantly associated with improvements in plant growth parameters, soil nutrient content, and rhizosphere microbial diversity (Figure 2, Figure 3, Figure 4 and Figure 5, Tables S7–S14). While these multivariate relationships suggest potential synergies, the specific mechanistic pathways require further validation through controlled inoculation experiments.

Strong statistical associations were observed between soil nutrient content and blueberry seedling survival rates (Figure 5, Tables S8–S14), consistent with previous reports on soil-plant interactions [44]. Similarly, covariation patterns emerged between rhizosphere microbial diversity and seedling survival, though the directionality of these relationships remains to be elucidated.

Plant height is influenced by environmental factors such as fertilization, light, and soil conditions [45]. These factors can either promote or limit plant growth within a certain range, although their effects may not always be pronounced [46]. Our results indicated that most soil nutrient contents, except for total nitrogen content (TN) and total phosphorus hydrolyzable content (TP), showed extremely significant or significant correlations with the plant height of blueberry seedlings (Figure 5, Tables S9 and S12). Among microbial diversity indices, only the Shannon index for bacteria exhibited a significant correlation with plant height (Figure 5, Tables S9 and S12).

Soil nutrient content and microorganisms interact closely, forming complex relationships [47]. Bacterial diversity indices were extremely significantly or significantly correlated with organic carbon content (OC), total nitrogen content (TN), and hydrolyzable nitrogen content (HN) (Figure 5, Tables S9 and S12). In contrast, nearly all soil nutrient contents showed extremely significant or significant correlations with fungal diversity indices (Figure 5, Tables S10 and S13).

Based on the above analyses, PGPR-treated substrates significantly influenced plant growth characteristics, soil nutrient content, and rhizosphere microbial diversity (Table S14). Plant growth characteristics were strongly enhanced by soil nutrient levels, and a certain correlation was observed between the rhizosphere microbial community and plant growth performance (Table S14). Additionally, soil nutrient content showed a moderate correlation with bacterial diversity and a strong correlation with fungal diversity (Table S14).

## 5. Conclusions

PGPR treatment was associated with accelerated SMS decomposition and improved blueberry growth. When mixed with nutrient soil at a 1:4 ratio, the PGPR-treated substrate increased seedling survival rates and chlorophyll content compared to the control. The improved plant growth was accompanied by enhanced soil nutrient availability, as shown by the significant increases in TN, HN, and AP. Furthermore, the result of high throughput sequencing showed that PGPR-treated substrates altered the microbial community composition in the blueberry rhizosphere. Future research should explore the application of these PGPR strains to other berry crops, identify the specific microbial taxa affected by the treated substrate, and optimize culture process conditions for different types of organic waste materials. These investigations would further validate the practical utility of PGPR-mediated waste conversion for sustainable horticultural production.

## Data Availability

The original contributions presented in this study are included in the article/Supplementary Materials. Further inquiries can be directed to the corresponding author.

## Supplementary Materials

The following supporting information can be downloaded at: https://www.mdpi.com/article/10.3390/microorganisms13040932/s1, Figure S1: Network analysis of multivariate relationships among PGPR functional traits, blueberry growth performance, soil nutrients, and rhizosphere microbiota; Table S1: The plant growth-promoting capabilities of PGPR strains; Table S2: Mean concentrations of nutritive elements and pH values (±standard deviation, SD) in SMS incubated with PGPR T1. Table S3: Mean concentrations of nutritive elements and pH values (#xB1; standard deviation, SD) in SMS incubated with PGPR strain T2. Table S4: Mean concentrations of nutritive elements and pH values (±standard deviation, SD) in SMS incubated with PGPR strain T3. Table S5: Mean concentrations of nutritive elements and pH values (±standard deviation, SD) in TC1. Table S6: Mean concentrations of nutritive elements and pH values (±standard deviation, SD) in TC2. Table S7: Survival rates of blueberry seedlings after 45-day transplantation under different treatments. Table S8: Kendall’s correlation analysis of growth-promoting characteristics of PGPR strains with plant growth characteristics, soil element content, and rhizosphere soil microbial diversity; Table S9: Kendall’s correlation analysis of plant growth characteristics with soil element content and rhizosphere soil microbial diversity; Table S10: Kendall’s correlation analysis of soil element content with rhizosphere soil microbial diversity; Table S11: Spearman’s correlation analysis of growth-promoting characteristics of PGPR strains with plant growth characteristics, soil element content, and rhizosphere soil microbial diversity; Table S12: Spearman’s correlation analysis of plant growth characteristics with soil element content and rhizosphere soil microbial diversity; Table S13: Spearman’s correlation analysis of soil element content with rhizosphere soil microbial diversity. Table S14: Eigenvector of three principal components of growth-promoting characteristics of PGPR strains with plant growth characteristics, soil element content, and rhizosphere soil microbial diversity.
