# Supplementary material for: Effects of Spent Mushroom Substrate Treated with Plant Growth-Promoting Rhizobacteria on Blueberry Growth and Soil Quality
Source: Microorganisms. 2025 Apr 17;13(4):932. doi: 10.3390/microorganisms13040932 (PMC12029275; doi:10.3390/microorganisms13040932)
Supplement: Supplementary file 1 [file microorganisms-13-00932-s001.zip › microorganisms-3541628-supplementary.pdf]

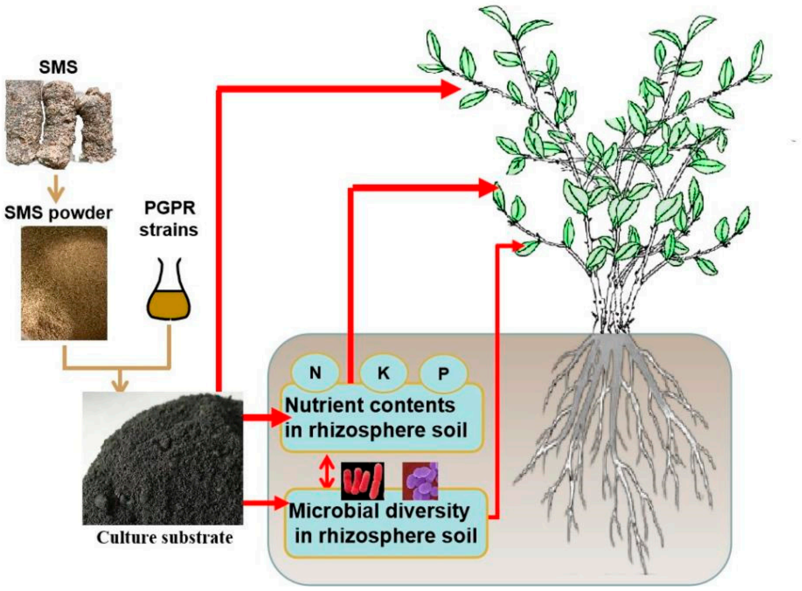

Figure S1. Network analysis of multivariate relationships among PGPR functional traits, blueberry growth performance, soil nutrients, and rhizosphere microbiota.

Notes: Red arrows indicate correlations, with arrow thickness proportional to correlation strength. Solid arrows represent direct interactions, while dashed arrows denote indirect interactions. Yellow arrows indicate processes involved in the production of the culture substrate.

Table S1. The plant growth promoting capabilities of PGPR strains

Scheme		T1	T2	T3
Accession number		MW590634	MW590630	MW590641
Sequence Length (bp)		909	999	999
Related type strain		*Pantoea* sp.	*Erwinia* sp.	*Serratia* sp.
Concentrations of phosphorus in liquid medium (mg/L)		3.23 ± 0.04	3.43 ± 0.39	2.72 ± 0.12
Concentrations of auxin in liquid medium (mg/L)		100.98 ± 5.49	111.59 ± 7.46	96.27 ± 6.80
The colony diameters of PGPR strains in different medium (cm)	Silicate bacteria medium	0.50 ± 0.03	0.43 ± 0.06	0.51 ± 0.02
	A Sugai's medium	0.43 ± 0.02	0.47 ± 0.02	0.45 ± 0.00
Final pH of fermentation solution		3.5	4.0	3.5

Table S2. Mean concentrations of nutritive elements and pH values (± standard deviation, SD) in SMS incubated with PGPR T1

Time (days)	OC（g/kg）	TN（g/kg）	HN（g/kg）	TP（g/kg）	AP（g/kg）	TK（g/kg）	AK（g/kg）	pH
0	419.75 ± 10.09a	13.58 ± 0.32a	1.42 ± 0.00a	4.01 ± 0.45a	2.13 ± 0.00a	7.02 ± 0.30a	2.84 ± 0.01a	5.50 ± 0.20a
3	426.50 ± 11.06b	16.58 ± 0.40b	1.62 ± 0.06b	4.80 ± 0.53b	3.59 ± 0.05b	7.55 ± 0.40b	3.33 ± 0.03b	5.50 ± 0.50a
9	426.49 ± 4.23b	16.04 ± 0.25b	1.52 ± 0.00c	4.76 ± 0.05b	3.54 ± 0.09b	7.83 ± 0.71c	3.50 ± 0.05c	5.63 ± 0.75b
27	432.74 ± 1.83c	13.88 ± 0.30c	1.96 ± 0.01d	4.65 ± 0.09c	3.58 ± 0.00b	7.88 ± 0.89c	3.46 ± 0.07c	5.69 ± 0.81c
54	439.12 ± 2.83d	16.02 ± 0.24b	1.99 ± 0.05d	4.77 ± 0.28b	3.84 ± 0.01c	8.28 ± 0.34d	3.96 ± 0.02d	5.78 ± 0.64d
90	434.75 ± 0.93e	15.77 ± 0.36d	2.09 ± 0.02e	4.50 ± 0.77d	3.55 ± 0.02b	8.08 ± 0.85e	4.06 ± 0.02e	5.59 ± 0.72a
135	449.77 ± 2.03f	14.97 ± 0.10e	1.99 ± 0.05d	4.64 ± 0.27c	3.25 ± 0.04d	7.93 ± 0.88d	3.63 ± 0.05f	5.50 ± 0.91a
180	439.25 ± 2.40d	14.34 ± 0.27f	1.85 ± 0.02f	4.33 ± 0.07e	2.27 ± 0.06e	7.54 ± 0.72b	3.61 ± 0.02f	5.52 ± 0.43a

Table S3. Mean concentrations of nutritive elements and pH values (± standard deviation, SD) in SMS incubated with PGPR strain T2.

Time (days)	OC（g/kg）	TN（g/kg）	HN（g/kg）	TP（g/kg）	AP（g/kg）	TK（g/kg）	AK（g/kg）	pH
0	419.35 ± 7.01a	13.60 ± 0.45a	1.46 ± 0.07a	4.00 ± 0.13a	2.08 ± 0.01a	6.92 ± 0.30a	2.82 ± 0.00a	5.50 ± 0.30a
3	420.13 ± 11.01b	16.57 ± 0.58b	1.69 ± 0.01b	5.17 ± 0.14b	3.93 ± 0.11b	7.73 ± 0.12b	3.13 ± 0.01b	5.54 ± 0.75a
9	428.74 ± 21.81c	14.58 ± 1.57c	1.64 ± 0.05b	4.50 ± 0.06c	3.54 ± 0.12c	7.67 ± 0.45c	2.86 ± 0.05a	5.60 ± 0.23a
27	450.93 ± 7.26d	14.29 ± 0.56d	1.96 ± 0.11c	4.04 ± 0.53a	3.54 ± 0.05d	7.00 ± 0.77a	3.73 ± 0.09c	5.70 ± 0.76a
54	453.83 ± 3.22e	15.97 ± 0.45e	2.31 ± 0.09d	4.94 ± 0.13d	3.65 ± 0.09d	8.19 ± 0.85d	3.92 ± 0.07d	5.67 ± 0.63a
90	448.57 ± 13.55f	16.18 ± 0.10f	2.21 ± 0.06e	4.91 ± 0.16d	3.57 ± 0.08d	8.19 ± 0.56d	3.86 ± 0.11e	5.70 ± 0.82a
135	458.04 ± 5.22g	15.28 ± 0.63g	1.96 ± 0.12c	5.11 ± 0.48b	3.30 ± 0.19e	7.78 ± 0.12b	3.40 ± 0.25f	5.52 ± 0.46a
180	448.50 ± 9.01f	14.95 ± 0.06h	1.92 ± 0.09c	4.73 ± 0.18e	2.21 ± 0.06f	7.47 ± 0.78e	3.35 ± 0.12f	5.53 ± 0.19a

Table S4. Mean concentrations of nutritive elements and pH values (± standard deviation, SD) in SMS incubated with PGPR strain T3.

Time (days)	OC（g/kg）	TN（g/kg）	HN（g/kg）	TP（g/kg）	AP（g/kg）	TK（g/kg）	AK（g/kg）	pH
0	420.00 ± 11.09a	13.56 ± 0.71a	1.52 ± 0.06a	4.03 ± 0.05a	2.10 ± 0.22a	6.94 ± 0.22a	2.85 ± 0.01a	5.50 ± 0.10a
3	419.32 ± 1.56a	15.83 ± 0.06b	5.12 ± 0.21b	7.46 ± 0.02b	1.56 ± 0.04b	6.47 ± 0.09b	3.07 ± 0.02b	5.69 ± 0.81b
9	419.27 ± 1.94a	15.80 ± 0.61b	4.77 ± 0.09c	7.81 ± 0.03c	1.58 ± 0.04b	6.70 ± 0.05c	3.40 ± 0.07c	5.78 ± 0.64c
27	433.55 ± 4.84b	13.51 ± 0.02a	4.41 ± 0.08d	7.58 ± 0.02b	1.62 ± 0.00c	6.85 ± 0.07d	3.69 ± 0.11d	5.59 ± 0.72a
54	435.89 ± 1.48b	15.96 ± 0.02c	5.16 ± 0.04b	8.02 ± 0.17d	2.37 ± 0.04d	6.90 ± 0.09a	3.96 ± 0.04e	5.67 ± 0.63b
90	428.83 ± 2.13c	15.86 ± 1.19c	5.03 ± 0.01e	8.41 ± 1.09e	2.26 ± 0.02e	6.39 ± 0.07e	3.87 ± 0.07f	5.71 ± 0.82b
135	438.81 ± 6.26d	15.06 ± 0.16d	4.68 ± 0.08f	7.84 ± 0.07c	2.15 ± 0.00a	6.39 ± 0.05e	3.77 ± 0.03d	5.59 ± 0.72a
180	428.04 ± 5.73c	14.60 ± 0.18e	4.44 ± 0.10d	7.51 ± 0.01b	2.05 ± 0.01a	6.38 ± 0.08e	3.64 ± 0.04d	5.50 ± 0.91a

Table S5. Mean concentrations of nutritive elements and pH values (± standard deviation, SD) in TC1.

Time (days)	OC（g/kg）	TN（g/kg）	HN（g/kg）	TP（g/kg）	AP（mg/kg）	TK（g/kg）	AK（g/kg）	pH
0	322.59 ± 19.07a	7.99 ± 0.00a	0.48 ± 0.04a	1.20 ± 0.13a	25.18 ± 3.83a	13.11 ± 0.22a	0.20 ± 0.00a	5.50 ± 0.10a
3	323.40 ± 10.00a	8.01 ± 0.03a	0.47 ± 0.02a	1.19 ± 0.14a	25.23 ± 2.55a	13.17 ± 0.25a	0.21 ± 0.01a	5.60 ± 0.11a
9	321.79 ± 5.02a	8.00 ± 0.00a	0.49 ± 0.03a	1.18 ± 0.15a	25.25 ± 2.87a	13.09 ± 0.33a	0.20 ± 0.00a	5.58 ± 0.12a
27	322.98 ± 13.42a	7.99 ± 0.04a	0.47 ± 0.00a	1.20 ± 0.11a	24.98 ± 4.57a	13.01 ± 0.42a	0.21 ± 0.03a	5.50 ± 0.17a
54	320.44 ± 17.33a	8.01 ± 0.02a	0.49 ± 0.04a	1.19 ± 0.17a	25.63 ± 1.83a	13.03 ± 0.75a	0.21 ± 0.00a	5.61 ± 0.10a
90	319.35 ± 11.95a	8.02 ± 0.03a	0.48 ± 0.03a	1.20 ± 0.08a	24.77 ± 3.54a	13.19 ± 0.84a	0.20 ± 0.03a	5.52 ± 0.10a
135	320.56 ± 12.27a	8.00 ± 0.00a	0.49 ± 0.02a	1.21 ± 0.10a	26.15 ± 1.23a	13.11 ± 0.78a	0.20 ± 0.06a	5.57 ± 0.13a
180	318.90 ± 13.76a	8.03 ± 0.06a	0.47 ± 0.03a	1.19 ± 0.09a	25.73 ± 2.83a	13.10 ± 0.17a	0.20 ± 0.00a	5.54 ± 0.09a

Table S6. Mean concentrations of nutritive elements and pH values (± standard deviation, SD) in TC2.

**Time (days)**	**OC（g/kg）**	**TN（g/kg）**	**HN（g/kg）**	**TP（g/kg）**	**AP（mg/kg）**	**TK（g/kg）**	**AK（g/kg）**	**pH**
0	349.88 ± 5.61a	8.29 ± 0.20a	0.52 ± 0.00a	1.50 ± 0.09a	567.50 ± 56a	13.37 ± 0.13a	0.28 ± 0.00a	5.62 ± 0.10a
3	352.76 ± 7.21a	8.13 ± 0.32a	0.51 ± 0.01a	1.54 ± 0.10a	534.50 ± 32a	13.02 ± 0.44a	0.30 ± 0.02a	5.65 ± 0.50a
9	354.33 ± 9.61a	8.20 ± 0.17a	0.52 ± 0.00a	1.51 ± 0.08a	570.69 ± 79a	13.89 ± 0.35a	0.29 ± 0.02a	5.60 ± 0.32a
27	345.21 ± 8.53a	8.10 ± 0.30a	0.51 ± 0.02a	1.58 ± 0.12a	563.44 ± 32a	13.65 ± 0.45a	0.28 ± 0.01a	5.70 ± 0.20a
54	348.77 ± 3.23a	8.33 ± 0.28a	0.51 ± 0.01a	1.50 ± 0.04a	555.54 ± 76a	13.62 ± 0.72a	0.27 ± 0.03a	5.60 ± 0.40a
90	349.20 ± 7.63a	8.12 ± 0.26a	0.53 ± 0.01a	1.49 ± 0.13a	547.50 ± 89a	13.22 ± 0.45a	0.28 ± 0.01a	5.72 ± 0.22a
135	348.90 ± 4.51a	8.18 ± 0.09a	0.51 ± 0.03a	1.50 ± 0.15a	573.44 ± 46a	13.87 ± 0.62a	0.27 ± 0.02a	5.68 ± 0.42a
180	350.33 ± 6.45a	8.25 ± 0.10a	0.52 ± 0.01a	1.58 ± 0.19a	560.88 ± 29a	13.66 ± 0.45a	0.28 ± 0.02a	5.74 ± 0.33a

Note: OC:The organic carbon contents, TN: total nitrogen contents, HN: hydrolysable nitrogen contents,TP: total phosphorous contents, AP: available phosphorous contents, TK: total potassium contents, and AK: vailable potassium contents.

Data expressed as mean ± SD of three replicates per treatment.

Different lowercase letters above bars indicate statistically significant differences (Tukey's HSD test, $p < 0.05$).

Underline the maximum value in each column.

Table S7. Survival rates of blueberry seedlings after 45-day transplantation under different treatments

	TC1	TC2	T1	T2	T3
Pot A	56.00%	56.00%	72.00%	80.00%	80.00%
Pot B	64.00%	60.00%	84.00%	76.00%	76.00%
Pot C	52.00%	64.00%	80.00%	84.00%	80.00%
Survival Rate (%)	57.33% ± 6.11%	60.00%±4.00%	78.67%±6.11%	80.00%±4.00%	78.67%±2.30%

Notes:

Treatment groups:T1: SMS decomposed with PGPR strain T1, T2: SMS decomposed with PGPR strain T2, T3: SMS decomposed with PGPR strain T3; Control groups: TC1: Nutrient soil alone (no SMS), TC2: Non-decomposed SMS mixed with nutrient soil (1:4 ratio).

All values are mean ± SD of triplicate experiments.

Table S8. Kendall's correlation analysis of growth promoting characteristics of PGPR strains with plant growth characteristics, soil element content, and rhizosphere soil microbial diversity.

Capacity of strains	Survival rate	Plant height	Chl	OCC	TNC	HNC	TPHC	APHC	TPOC	APOC	Simpson B	Shannon B	Simpson P	Shannon P
Phosphorus	**0.601****	0.356*	**0.459****	0.307	**0.533****	**0.611****	**0.638****	**0.647****	**0.721****	**0.523****	0.310	0.277	**0.502****	**0.677****
Auxin	**0.586****	0.399*	0.422*	0.317	**0.518****	**0.619****	**0.640****	**0.609****	**0.693****	**0.566****	0.256	0.287	**0.510****	**0.638****
Silicate decomposition	**0.522****	**0.494****	**0.565****	**0.761****	**0.565****	**0.669****	0.321	**0.451****	**0.507****	**0.513****	**0.576****	**0.532****	0.385*	0.424*
Nitrogen fixation	**0.538****	0.322	**0.710****	**0.466****	**0.724****	**0.634****	**0.606****	0.453*	**0.559****	0.395*	**0.532****	0.439*	**0.537****	**0.710****

Table S9. Kendall's correlation analysis of plant growth characteristics with soil element content and rhizosphere soil microbial diversity.

	OCC	TNC	HNC	TPHC	APHC	TPOC	APOC	Simpson B	Shannon B	Simpson P	Shannon P
Survival rate	**0.552****	**0.456****	**0.560****	**0.514****	**0.545****	**0.516****	**0.479****	0.389*	0.400*	0.304	**0.525****
Plant height	**0.511****	0.149	**0.469****	0.233	**0.462****	0.400*	**0.565****	0.402*	**0.466****	0.063	0.253
Chl	**0.521****	**0.745****	0.372*	**0.450****	0.260	**0.453****	0.206	**0.563****	0.339*	**0.529****	**0.547****

Table S10. Kendall's correlation analysis of soil element content with rhizosphere soil microbial diversity.

	Simpson B	Shannon B	Simpson P	Shannon P
OCC	**0.515****	**0.513****	0.396*	**0.426****
TNC	**0.531****	0.321	**0.567****	**0.713****
HNC	0.401*	0.374*	0.352*	**0.491****
TPHC	0.282	0.322	**0.518****	**0.634****
APHC	0.216	0.219	0.283	**0.430****
TPOC	0.304	0.188	**0.563****	**0.581****

APOC	0.258	0.334*	0.228	0.311

Note:

Chl: total chlorophyll, OCC:The organic carbon contents, TNC: total nitrogen contents, HNC: hydrolysable nitrogen contents,TPHC: total phosphorous contents, APHC: available phosphorous contents, TPOC: total potassium contents, and APOC: available potassium contents. Simpson B and Shannon B were α diversity index of rhizosphere bacteria, and Simpson P and Shannon P were α diversity index of rhizosphere fungi.

** was as significantly associated at 0.01 level (bilateral).

* was as significantly associated at 0.05 level (bilateral).

Table S11. Spearman's correlation analysis of growth promoting characteristics of PGPR strains with plant growth characteristics, soil element content, and rhizosphere soil microbial diversity.

Capacity of strains	Survival rate	Plant height	Chl	OCC	TNC	HNC	TPHC	APHC	TPOC	APOC	Simpson B	Shannon B	Simpson P	Shannon P
Phosphorus	**0.806****	0.505*	**0.658****	0.556*	**0.717****	**0.814****	**0.782****	**0.810****	**0.860****	**0.676****	0.479*	0.426	**0.653****	**0.858****
Auxin	**0.791****	0.530*	**0.642****	0.556*	**0.703****	**0.823****	**0.782****	**0.801****	**0.851****	**0.723****	0.469*	0.426	**0.666****	**0.813****
Silicate decomposition	**0.715****	**0.649****	**0.759****	**0.896****	**0.740****	**0.814****	0.462*	**0.615****	**0.692****	**0.681****	**0.731****	**0.668****	0.539*	**0.639****
Nitrogen fixation	**0.719****	0.455*	**0.865****	**0.640****	**0.868****	**0.778****	**0.724****	**0.609****	**0.734****	0.557*	**0.673****	0.549*	**0.650****	**0.866****

Table S12. Spearman's correlation analysis of plant growth characteristics with soil element content and rhizosphere soil microbial diversity.

	OCC	TNC	HNC	TPHC	APHC	TPOC	APOC	Simpson B	Shannon B	Simpson P	Shannon P
Survival rate	**0.732****	**0.665****	**0.727****	**0.677****	**0.720****	**0.755****	**0.661****	0.539*	0.504*	0.515*	**0.720****

Plant height	**0.712****	0.322	**0.647****	0.389	**0.647****	0.545*	**0.709****	0.535*	**0.638****	0.120	0.397
Chl	**0.748****	**0.907****	**0.609****	**0.607****	0.483*	**0.664****	**0.447***	**0.755****	**0.535***	**0.731****	**0.771****

Table S13. Spearman's correlation analysis of soil element content with rhizosphere soil microbial diversity.

	Simpson B	Shannon B	Simpson P	Shannon P
OCC	**0.715****	**0.672****	0.538*	**0.654****
TNC	**0.687****	0.439	**0.778****	**0.852****
HNC	**0.575****	0.543*	0.495*	**0.692****
TPHC	0.457*	0.445*	**0.628****	**0.817****
APHC	0.373	0.339	**0.427**	**0.650****
TPOC	0.512*	0.351	**0.728****	**0.757****
APOC	0.443	0.487*	0.332	0.467*

Note:

Chl: total chlorophyll, OCC:The organic carbon contents, TNC: total nitrogen contents, HNC: hydrolysable nitrogen contents,TPHC: total phosphorous contents, APHC: available phosphorous contents, TPOC: total potassium contents, and APOC: available potassium contents. Simpson B and Shannon B were α diversity index of rhizosphere bacteria, and Simpson P and Shannon P were α diversity index of rhizosphere fungi.

** was as significantly associated at 0.01 level (bilateral).

* was as significantly associated at 0.05 level (bilateral).

Table S14. Eigenvector of three principal components of growth promoting characteristics of PGPR strains with plant growth characteristics, soil element content, and rhizosphere soil microbial diversity.

	Principal component		
	1	2	3
Phosphorus	**0.961**	0.030	0.026
Auxin	**0.970**	0.072	-0.014
Silicate decomposition	**0.965**	0.157	0.055
Nitrogen fixation	**0.970**	0.134	-0.041
Survival rate	**0.924**	0.051	0.124
Plant height	0.611	-0.061	0.720
Chl	**0.867**	0.219	-0.210
OCC	0.844	-0.085	0.093
TNC	**0.929**	0.102	-0.272
HNC	**0.950**	0.012	0.128
TPHC	0.821	-0.421	-0.140
APHC	0.824	-0.425	0.076
TPOC	0.767	-0.272	-0.047
APOC	0.787	-0.415	0.336
Simpson B	0.630	0.630	-0.123
Shannon B	0.636	0.560	0.306
Simpson P	0.686	-0.198	-0.608
Shannon P	**0.893**	-0.004	-0.246

Note: Determinant factors were chosen when the absolute value is greater than or equal to 0.850.

Chl: total chlorophyll, OCC:The organic carbon contents, TNC: total nitrogen contents, HNC: hydrolysable nitrogen contents,TPHC: total phosphorous contents, APHC: available phosphorous contents, TPOC: total potassium contents, and APOC: available potassium contents. Simpson B and Shannon B were α diversity index of rhizosphere bacteria, and Simpson P and Shannon P were α diversity index of rhizosphere fungi
